# Assessing the impact of climate change on verticillium wilt and the implications for cotton production in Australia

**DOI:** 10.1007/s00484-025-03100-5

**Published:** 2026-02-10

**Authors:** Karen A. Kirkby, Jane M. Kelley, Bethany Ellis, James R. Lawson, Christopher Nunn, Rebecca O. Darbyshire, Joanna Pardoe

**Affiliations:** 1 Australian Cotton Research Institute, NSW Department of Primary Industries and Regional Development, Narrabri, New South Wales Australia; 2 Climate Branch, NSW Department of Primary Industries and Regional Development, Narrabri, New South Wales Australia; 3 CSIRO Agriculture and Food, Canberra, Australian Capital Territory Australia

**Keywords:** Verticillium dahliae, Agricultural production, Strain, Modelling, Multi-criteria analysis

## Abstract

Climate change poses significant challenges for agricultural production, potentially altering crop distribution, productivity, and the prevalence of plant diseases. This study focuses on the fungus *Verticillium dahliae* which causes disease in over 400 plant species, significantly impacting cotton in most major cotton producing countries. We investigate how climate suitability for *V. dahliae* could change in the future, using New South Wales (NSW), Australia, as a case study. Our research examines the interplay between factors affecting the prevalence of *V. dahliae* infection, including fungal strain, temperature and rainfall. Using a 1992multi-criteria analysis approach, we evaluated climate suitability for *V. dahliae* under both historical and projected mid-21st century future climate conditions. This method combines peer-reviewed evidence with expert knowledge to assess potential impacts. Our findings suggest that climate change is likely to alter the number of months that are suitable for *V. dahliae* growth and potentially shift the distribution of fungal strains across NSW. Our modelling shows that the more aggressive defoliating strain is likely to become more prevalent in northern NSW, and the less aggressive non-defoliating strain is likely to become less prevalent, particularly in northern NSW. Our study provides valuable insights for agricultural planning and adaptation strategies in the face of climate change.

**Supplementary Information:**

The online version contains supplementary material available at 10.1007/s00484-025-03100-5.

## Introduction

Climate change future projections for Australia indicate significant increases in temperature and shifts in precipitation patterns (CSIRO and Bureau of Meteorology 2022; Pearce et al. 2007). These changes are likely to influence agricultural production, affecting productivity, quality and land use (Romeijn et al. 2014). The complexities of climate impacts on agriculture are multifaceted; for instance, while elevated temperatures may enhance plant growth, they can also promote the survival and germination of plant pathogens, leading to adverse outcomes. Such climate variations can influence both the distribution and productivity of essential food and fibre crops, along with the pathogens they carry (Burdon and Zhan 2020). Consequently, agricultural losses may arise directly from climate change affecting plant growth and indirectly from shifts in the prevalence of plant disease caused by these pathogens. One such pathogen is *Verticillium dahliae* Kleb., a soil-borne fungus responsible for the vascular disease Verticillium wilt, which affects over 400 plant species (European Food Safety Authority 2014). In cotton, this pathogen causes severe yield losses and declines in fibre quality (Kibalou et al. 2021) resulting in economic losses in food and fibre crop production (Pegg and Brady 2002; Subbarao et al. 1997).

The top ten cotton producing countries are India, China, United States, Pakistan, Brazil, Australia, Uzbekistan, Turkey, Turkmenistan and Burkin Faso producing more than 25 million tonnes of cotton worldwide annually (Khan et al. 2020). Cotton is a major crop in Australia, valued at $4.6 billion for the 2021/2022 season (Cotton Australia, 2022). The crop is predominantly grown as an irrigated crop, however large areas are grown dryland when climatic conditions are favourable. Verticillium wilt remains a major issue, with yield losses reported at up to 25% (Allen, 1992) and, even as high as 62% in specific fields (Holman et al. 2016). Verticillium wilt can be caused by six species: *V. dahliae*,* V. albo-atrum*,* V. nubilum*,* V. theobromae and V. tricorpus*. To date the only report of Verticillium wilt in cotton in Australia is caused by the *V. dahliae*. The arrival of Verticillium wilt caused by *V. dahliae* was first recorded in Australia in 1959 (Anon.) and later in 1967 (Evans and Paull 1967). To date, no chemicals are available to control Verticillium wilt in cotton anywhere in the world. Efforts to mitigate the impact of Verticillium wilt in each country rely on integrated pest management strategies. In Australia, these include selecting more disease-tolerant varieties, strategic irrigation scheduling, optimal use of nitrogen and potassium, timely harvesting, and rapid post-harvest incorporation of crop stubble.

The dynamics of *V. dahliae* and its impact on crop production are influenced by factors, including host susceptibility, pathogen inoculum density, virulence, dispersal mechanisms, and environment. Environmental conditions conducive to *V. dahliae* are likely to be altered by climate change (Requena-Mullor et al. 2020). Seasonal fluctuations can affect both the survival and spread of the pathogen. Following infection of the plant during the parasitic life stage, *V. dahliae* can cause wilting, leaf necrosis and even the death of plants. This is when the pathogen moves into a dormant life stage and generates long term survival structures known as microsclerotia. These microsclerotia are returned to the soil in the dying and dead plant tissue. Microsclerotia can remain viable in the soil for more than 14 years (Wilhelm 1955). This becomes the primary inoculum source for subsequent cotton growing seasons (Rowe and Powelson 2002). Their survival and germination patterns are closely tied to moisture conditions, with wet soil during dormancy promoting repeated germination, particularly in irrigated systems like cotton. However, the germination success of microsclerotia diminishes with successive wetting and drying cycles (Farley et al. 1971). The optimal temperature for microsclerotia germination is 20 °C, which initiates the transition back to the parasitic life stage and the onset of disease (Ben-Yephet and Pinkas 1977).

**Fig. 1:**
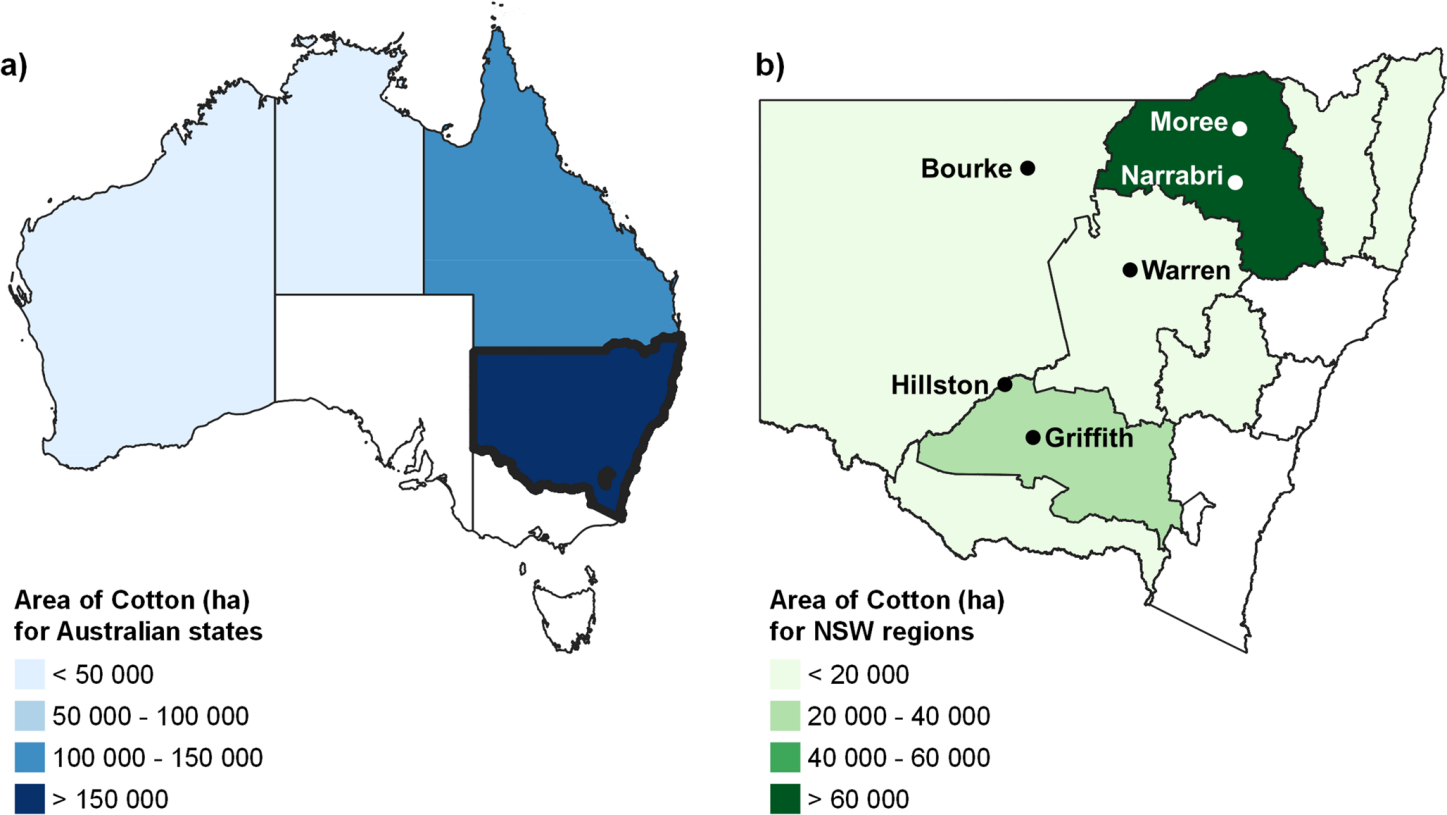
**a**) Irrigated and dryland cotton areas (ha) grown in Australia during the 2020-2021 season and **b**) cotton areas (ha) in NSW. (Data sourced from ABS Agricultural Commodities Estimates (2021)

The parasitic life stage of *V. dahliae* is divided into strains consisting of non-defoliating (ND) and defoliating (D) strains, also known as pathotypes, which can be further divided into vegetative compatibility groups (VCGs), often associated with virulence within hosts (Jiménez-Gasco et al. 2014). Internationally, in cotton, the D strain is highly virulent, causing leaf necrosis, defoliation and sometimes the death of plants, while the ND strain is generally less virulent. In Queensland (QLD) and New South Wales (NSW) cotton (Figure 1), both strains contribute to disease severity, with the ND VCG2A reported to cause significant damage, even surpassing the effects of the more virulent D VCG1A in certain regions (Dadd-Daigle et al. 2020).

Many early cotton farms in NSW were previously sheep grazing properties with a long history of common weeds, many known hosts to *V. dahliae* (Evans 1971). Following establishment in crops or weed hosts, the pathogen is primarily spread through soil movement by cultivation and movement in water. While optimum climatic conditions for *V. dahliae* growth under controlled settings have been extensively studied, the impact on the pathogen’s widespread geographic distribution remains less understood. Zhao et al. (2016) commented that in the global context of climate change, disease incidence will also be affected by elevated ozone, humidity, drought, and increased temperature.

Information about future changes in climate suitability of *V. dahliae* is vital knowledge for the Australian cotton industry. Australia’s cotton growing season lasts approximately six months, starting with planting in September/October and ending with picking in March/April. Given that early cotton growth stages are particularly susceptible to infection (Kirkby et al. 2013), any increase in pathogen suitability during this critical period could result in severe crop losses. It is essential to identify the timing and geographic scope of these changes to guide effective future management strategies. In Australia, there has been no specific research to determine the climate conditions that influence the distribution of *V. dahliae* or the impacts of future climate change. There are few studies utilising future climate future projections to investigate potential changes in *V. dahliae* occurrence. Zhang et al. (2024) found temperatures between the base growing temperature for cotton (15 °C) and the optimal infection threshold for Verticillium wilt (25 °C) reduced cotton yield. Interestingly, there was potential for yield increases with increased temperature, if there was no temperature effect on the *V. dahliae* infection. Requena-Mullor et al. (2020) investigated a range of rainfall and temperature climate drivers in the occurrence in olive-growing areas in southern Spain. A key finding being long-term climatic factors can explain the spatial patterns of *V. dahliae* in southern Spain. No other study has examined the changing climate’s role in dictating the suitability and geographic distribution of the Verticillium strains in Australian cotton-growing regions.

## Materials and methods

In this study, we developed a climate impact model to capture key bioclimatic relationships for the pathogenic and dormant life stages of *V. dahliae*. The objective of this study was to explore the impact of future climate change on climate suitability for both strains. The study adopted a flexible, transparent modelling approach known as ‘multi-criteria analysis’ (MCA) (Romeijn et al. 2016). This modelling approach enabled the synthesis of climate data, research literature and expert knowledge to produce spatial estimates of past and future climate suitability for *V. dahliae* across NSW.

### MCA model development and expert engagement

A multi-criteria analysis model was developed to capture the key climatic factors influencing the survival, growth and persistence of *V. dahliae*. The MCA model describes how *V. dahliae* responds to these climatic factors by specifying the climatic conditions that are suitable for the pathogen. The selection of climate variables for the MCA model was guided by research literature and expert knowledge through an iterative engagement process.

The MCA development process commenced with an initial MCA model being drafted. This involved identifying important life stages, and key climate variables and thresholds relating to them. The draft MCA model was then presented to a focus group of experts for refinement. The focus group, which included academics and industry extension officers, addressed knowledge gaps, especially differences between Australian and international strains (Dadd-Daigle et al. 2020, 2021, 2022) to enhance the MCA model. Each model component was then assigned an importance weight by consensus of the focus group and using the Analytical Hierarchy Process (AHP).

MCA models are structured as a hierarchical tree. The AHP assigns weightings using a pairwise comparison to calculate the relative importance of growth stages and climate variables at each level of the model hierarchy. These weightings reflect the contribution of each component to the successful growth of the pathogen. This AHP process (Romeijn et al. 2016; Saaty 1980, 1987, 1994) allows for the amalgamation of orthogonal or competing variables into an overarching climate suitability metric.

### Final MCA model for *Verticillium dahliae*

**Fig. 2:**
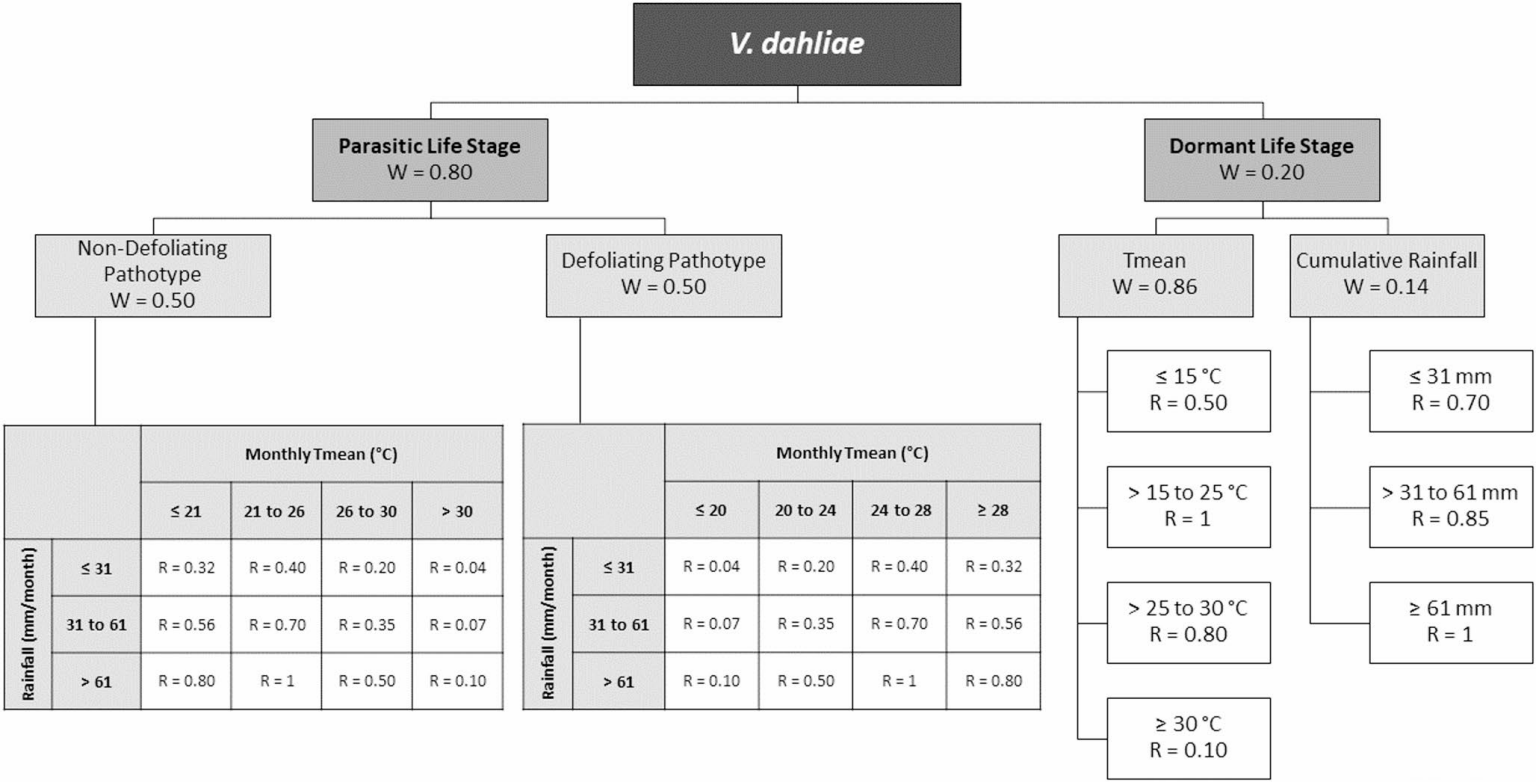
MCA model for V. dahliae. Weightings (W) are allocated for each variable and add up to 1 for each branch. Ratings (R) between 0 and 1 are assigned to indicate the influence of temperature and rainfall criterion on survival and reproduction, where 0 represents unfavourable climate conditions and 1 optimal climate conditions. Matrices are used for interacting variables for non-defoliating strain and defoliating strain. In the matrices monthly mean temperature categories are shown on the x-axis, and monthly rainfall categories are shown on the y-axis with rating parameters corresponding to each temperature/rainfall combination

The final MCA model integrates a combination of published literature and expert knowledge to inform and justify the structure and parameters of the *V. dahliae* MCA model (Figure 2). This combination of knowledge sources was critical to allow a model for the full life cycle of *V. dahliae* to be developed. While there are many published studies describing the parasitic life stage, detailing the differentiation of strains (Pérez-Artés et al. 2000; Usami 2013; Xu et al. 2012), there have been no studies characterising microsclerotia into strains in the dormant life stage. The MCA model described both the parasitic and dormant life stages, with the parasitic life stage being further divided into descriptions of the D and ND strains. No differentiation between strains was made for the dormant life stage, however. The parameters for both life stages were determined by identifying optimal temperature (from laboratory studies around the world) or rainfall thresholds stated for the pathogen from peer-reviewed literature. Data sources for the MCA model were categorised as published literature, modified data, expert experience or modified published literature (Supplementary Table I).

Climate suitability for the parasitic life stages of the MCA model was described by the interaction between temperature and rainfall, represented by matrices (Figure 2, Supplementary Table II). The temperature axis assigned climate suitability according to monthly mean temperature (calculated as the average of mean daily temperature, T_mean_) for a given month. The rainfall axis assigned climate suitability according to monthly rainfall. The ND and D strains required distinct thresholds and different temperature categories (Supplementary Table II). For the ND strain, the optimal temperature range was categorised as 21–25 °C and for the D strain, the optimal temperature range was categorised as 24–28 °C. Optimal rainfall was categorised as > 61 mm/month for both strains. Climate suitability for the temperature and rainfall components of the dormant life stage was calculated by transforming the monthly average daily T_mean_ data and the cumulative sum of daily rainfall.

The MCA model’s climate suitability ratings were established via normalisation functions that described significant ecoclimatic relationships (Holzkämper et al. 2013). These functions assigned unitless suitability values to categories of climate data, resulting in values between 0 and 1 for each month.

The weightings in the MCA model reflect each branch’s contribution to the pathogen’s overall climate sensitivity. The expert focus group deemed the parasitic life stage of higher importance (W = 0.8) than the dormant life stage (W = 0.2). The ND and D strains were equally weighted at 0.5 within the parasitic life stage. For the dormant life stage, the mean temperature influence was considered more important and assigned a weighting of 0.86, while the rainfall impact was weighted at 0.14.

### Historical and future climate data

The MCA model for *V. dahliae* used temperature and rainfall-based climate variables. Gridded historical data were sourced from the Australian Water Availability Project (AWAP), (Jones et al. 2009) at 0.05° resolution. Climate projection data were sourced from the ‘Climate Change in Australia: Application-Ready Dataset’ (CSIRO and Bureau of Meteorology 2015). The climate projection data included an ensemble of eight global climate models (GCMs) from CMIP5 selected as representing a range of plausible potential future climates across Australia (ACCESS1.0, CES1-CAM5, CNRM-CM5, GFDL-ESM2M, HadGEM2-CC, CanESM2, MIRCO5 and NorESM1-M). Two representative concentration pathways (RCPs) (Moss et al. 2010) were evaluated: RCP4.5 and RCP8.5. The RCP4.5 scenario describes an intermediate anthropogenic greenhouse-gas emissions scenario involving substantial emissions reductions in the latter half of the 21 st century. RCP8.5 is a high emissions scenario in which greenhouse gas emissions continue to rise throughout the 21 st century. The combinations of GCMs and RCPs meant that a total of 16 simulations were considered. The historical analyses use a baseline period of 30 years spanning 1981–2010, centred on 1995. Future climate suitability was estimated for the 30 years spanning 2036–2065, centred on 2050. The MCA model focuses exclusively on climate suitability and does not account for other factors affecting *V. dahliae*, like agricultural management decisions or extreme climate events. Climate variables like humidity, wind, soil characteristics, and pathogen inoculum levels were excluded due to their localised and variable nature. The MCA model assumes the presence of susceptible hosts.

### Calculating climate suitability

Climate suitability for *V. dahliae* was calculated by applying the MCA model to historical and projected climate data. Climate suitability can be extracted for any level of the MCA model structure, allowing assessment of climate impacts on either life stage or pathogen strain. Climate suitability focuses solely on how climate conditions support pathogen survival without accounting for other constraining factors, such as soil conditions or management decisions (Zhao et al. 2016). Thus, climate suitability amalgamates the pathogen’s exposure to climate conditions and its sensitivity to these conditions at a specific location. Uncertainties associated with projecting Earth’s climate systems into the future are carried through to the analyses of these results, however the use of confidence levels when assessing the change in climate suitability helps evaluate these uncertainties. The categorisation of these confidence levels highlights the consistency among GCMs and helps gauge their agreement in the future projections.

Historical climate suitability results are reported as the mean of the 30-year time series (1981–2010) over which the MCA model was run. Future projections of climate suitability are reported as an ensemble median of the eight GCM-specific time series (2036–2065) means. Change in climate suitability shows the difference between the historic climate suitability and the future projected climate suitability for both emissions scenarios. Confidence in the changing climate suitability was assessed using the ratio of the absolute value of median change across the eight GCMs to the standard deviation of climate suitability across the 8 GCMs. This ratio expresses the level of agreement of the MCA model outputs between the different GCMs. Higher ratios reflect greater confidence that all eight GCMs agree on the extent of change in climate suitability.

Spatial calculation of climate suitability from climate data was implemented in R (R Core Team 2024). Raster reclassification was used to transform climate data according to the defined rating parameters, and a weighted linear combination was used to sum the resulting rasters according to their assigned weightings. Calculations were performed using the *raster*,* terra*,* sp and rgeos* R packages (Bivand et al. 2017; Hijmans et al. 2015, 2022; Pebesma and Bivand 2005; Wickham 2011) and all figures were produced in R using ggplot2 and rasterVis packages (Lamigueiro and Hijmans 2023).

## Results

### Historical and future climate suitability

Maps were generated to represent historical and future climate suitability for both the overall MCA model and different life stages of *V. dahliae* for all 12 months of the year. These maps present climate suitability on a scale of 0 to 1, with 0 representing very low suitability in pale yellow to 1 being very high suitability shown in dark green. Change in suitability between the historic and future scenarios is shown on a scale from green (increase) to white (negligible) to purple (decrease). Confidence in the change is shown in shades of grey, with darker shades showing increased agreement between the GCMs.

Calendar plots display the monthly historic and future climate suitability values for key cotton growing regions in NSW, Australia, represented by the sites of Bourke, Moree, Narrabri, Warren, Hillston, and Griffith for both the RCP4.5 and RCP8.5 scenarios. The same colour scale as the maps is used to represent climate suitability. The values shown in the calendar plots are the median of values within a 10 km radius surrounding each site of interest. Within the calendar plots the columns represent months of the year starting from September (planting time) through to August the following season, with rows showing historical suitability, future suitability, change and confidence for each site of interest.

### Non-defoliating strain

**Fig. 3:**
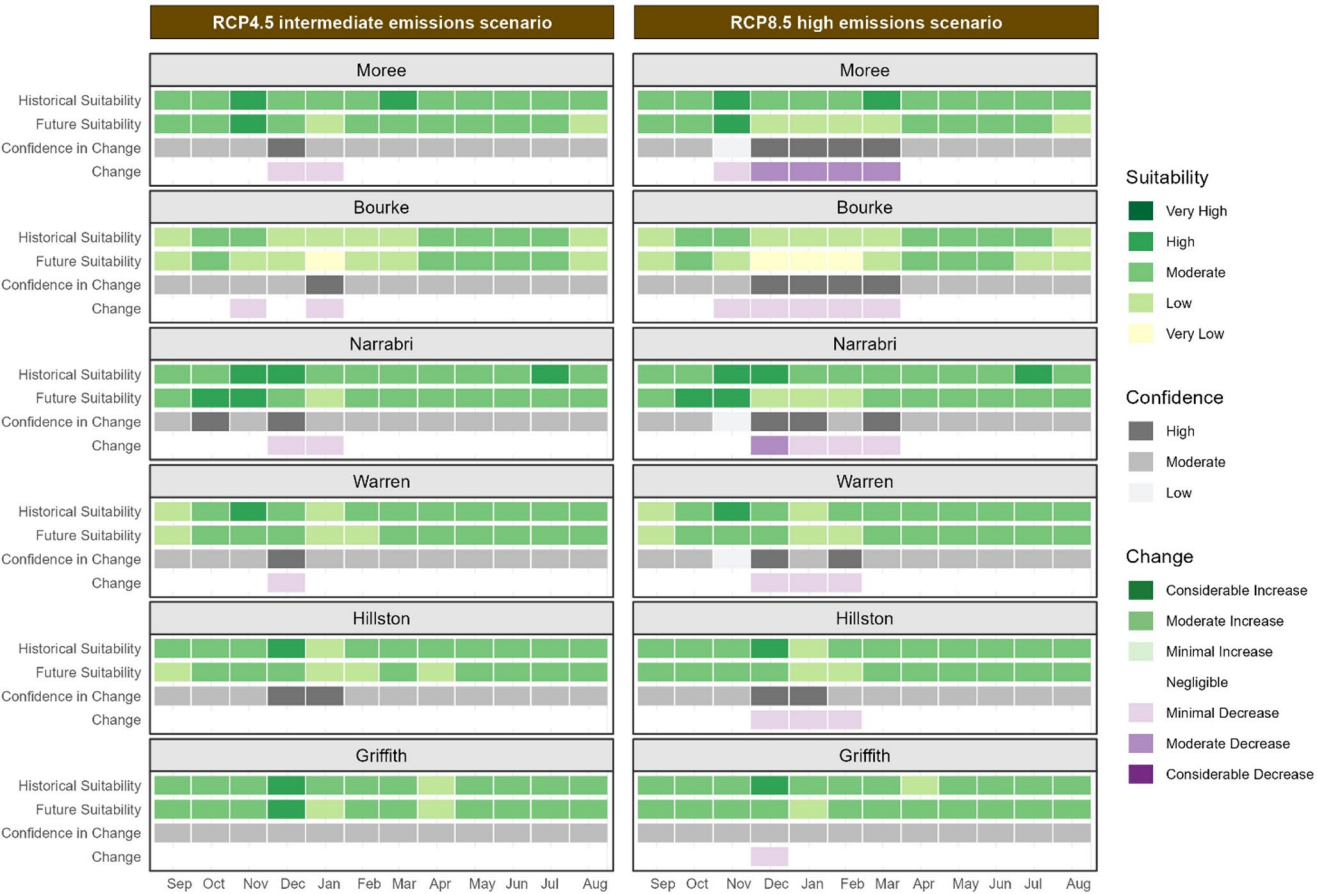
Calendar plot for the non-defoliating strain of V. dahliae during the cotton season in key cotton growing regions displaying the historical and future climate suitability, change in suitability and confidence in change for each site of interest. Two emissions scenarios are shown: the intermediate emissions scenario, RCP4.5, on the left and the high emissions scenario, RCP8.5, on the right. The climate suitability categories represent the median of values within a 10 km radius of each location

Future projections for future climate suitability of the non-defoliating (ND) strain of *V. dahliae* indicate likely changes across NSW cotton-growing regions by 2050 (Figure 3).

**Fig. 4:**
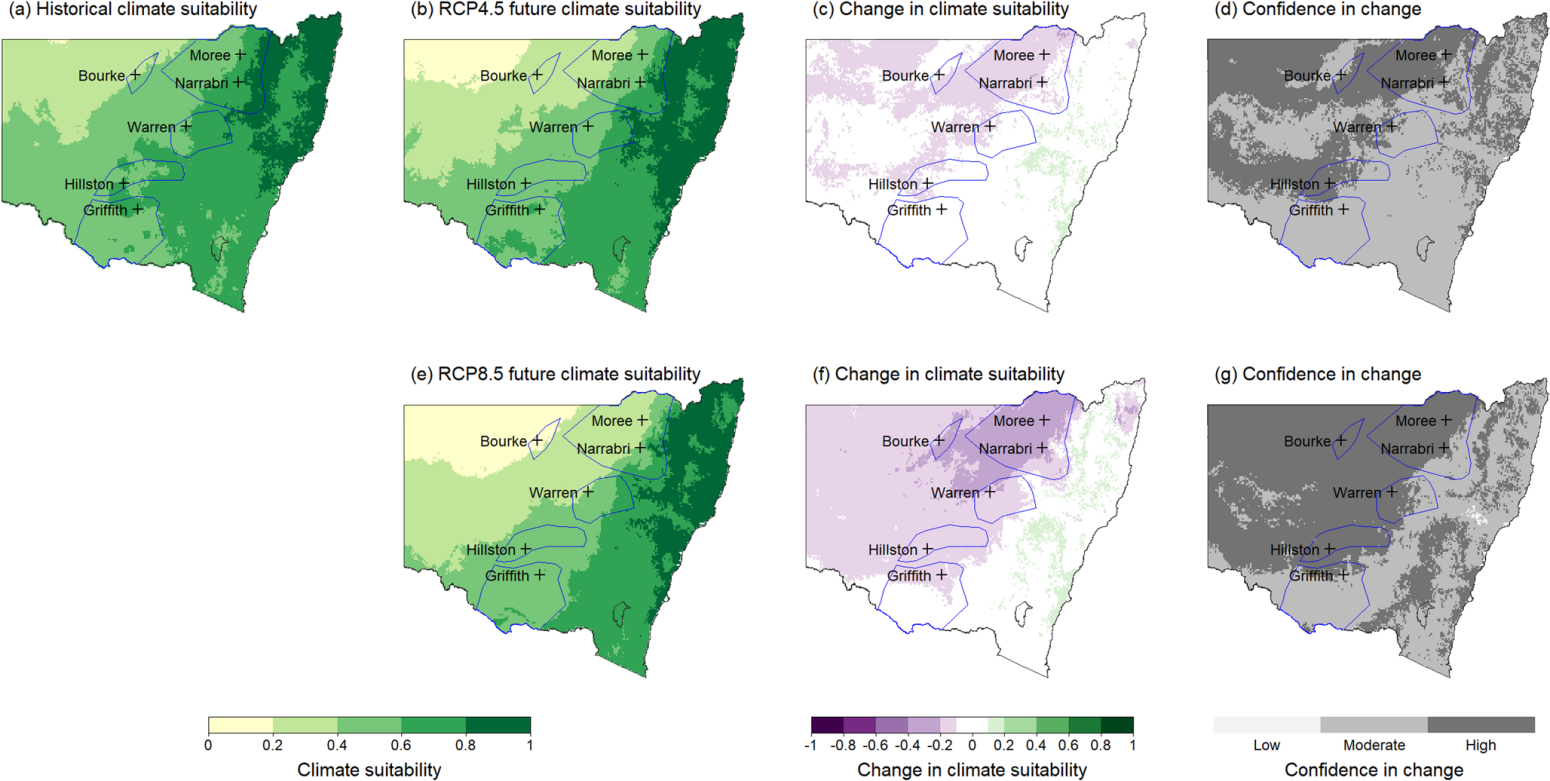
Map for December showing potential changes in climate suitability for the non-defoliating strain of V. dahliae across NSW cotton production areas (marked by blue polygons). **a**) Historical climate suitability across NSW, **b**) Future climate suitability under the intermediate emissions scenario RCP4.5, **c**) change in climate suitability under RCP4.5, **d**) confidence in change under RCP4.5. **e**) Future climate suitability under the high emissions scenario RCP8.5, **f**) change in climate suitability under RCP8.5, **g**) confidence in change under RCP8.5

Historically, the northern cotton growing regions in NSW, represented by Moree, Bourke, and Narrabri, have experienced moderate to high climate suitability for the ND strain. By 2050, climate suitability in these northern cotton-growing regions during summer months (December to February) is projected to decrease from high to moderate. This reduction is expected to be more pronounced under the high emissions scenario. The December maps (Figure 4) illustrate this declining mid-season climate suitability, particularly in the north of the state, under the high emissions scenario (Figure 4 and f). This shift is significant as Verticillium wilt symptoms in cotton crops typically becomes apparent during this period in NSW, with lower leaves exhibiting wilting, yellowing, necrosis, and defoliation.

Climate suitability for the ND strain in the Bourke cotton growing region has historically been lower than in other regions due to higher temperatures during the cotton growing season (September through to April). Climate suitability is projected to decrease to low or very low levels during summer by 2050. In the southern regions of Warren, Hillston, and Griffith, climate suitability for the ND strain has historically been moderate to high throughout the cotton growing season. Future projections suggest that climate suitability in these southern regions is likely to remain relatively stable under the intermediate emissions scenario. However, under the high emissions scenario, a minimal decrease in suitability is anticipated during the Australian summer months (December, January and February).

### Defoliating strain

**Fig. 5:**
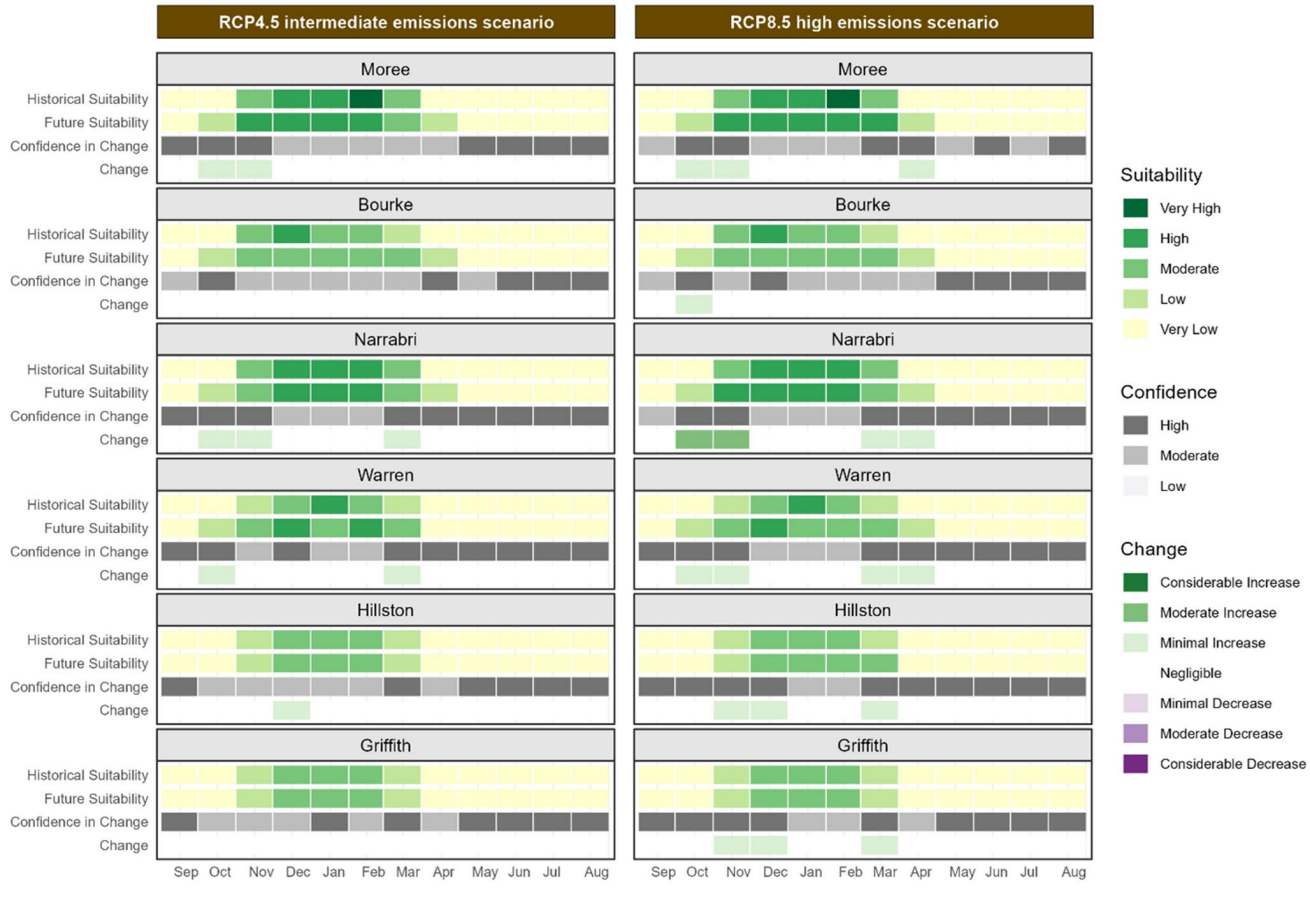
Calendar plot for the defoliating strain of *V. dahliae* during the cotton season in key cotton growing regions displaying the historical and future climate suitability, change in suitability and confidence in change for each site of interest. Two emissions scenarios are shown: the intermediate emissions scenario, RCP4.5, on the left and the high emissions scenario, RCP8.5, on the right. The climate suitability categories represent the median of values within a 10 km radius of each location

Climate suitability for the defoliating (D) strain of *V. dahliae* varies from low to very high across the NSW cotton-growing regions, with northern regions historically experiencing moderate to very high suitability (Figure 5).

**Fig. 6:**
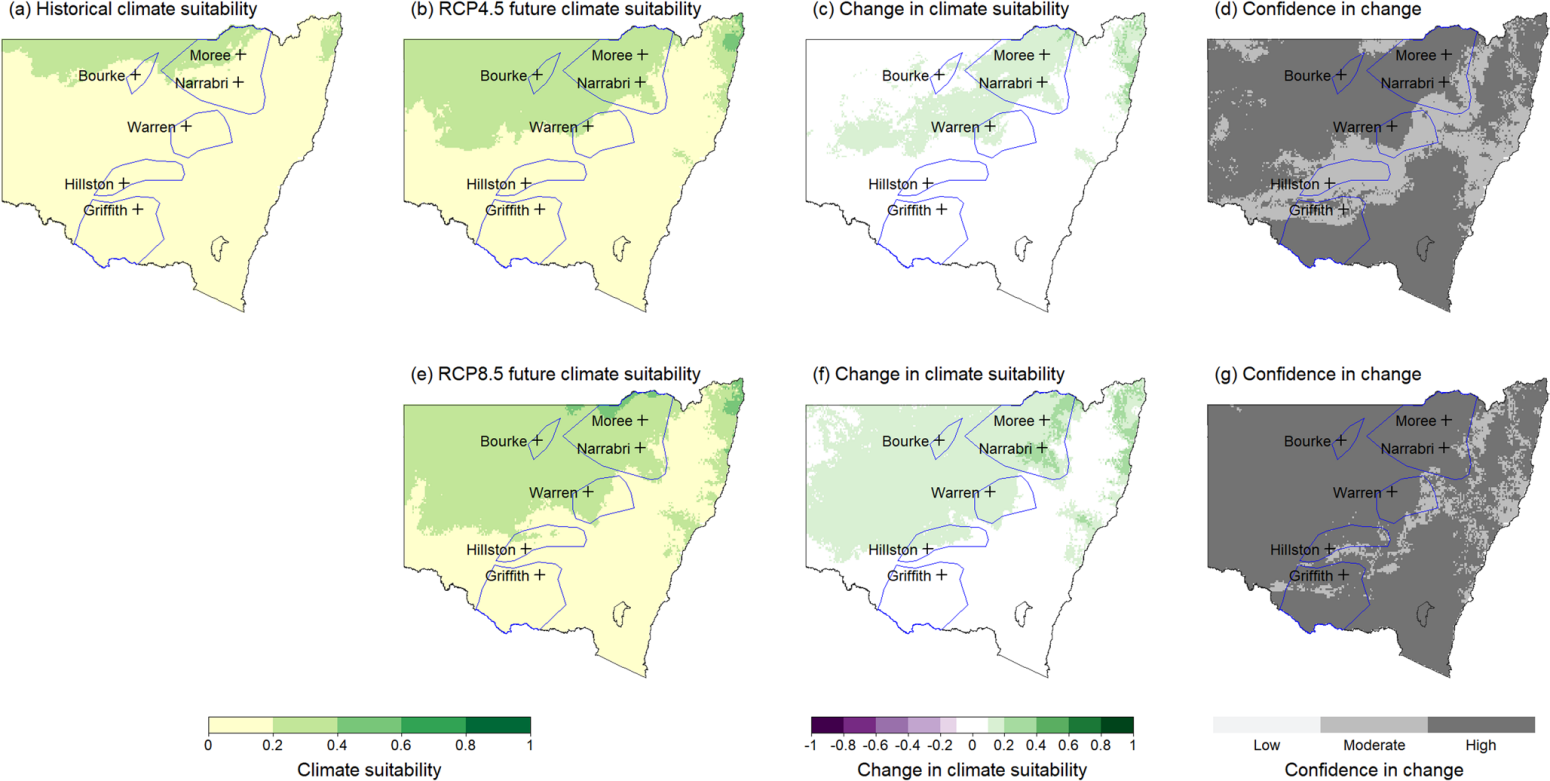
Output map for October showing potential changes in climate suitability for the defoliating strain of *V. dahliae* across NSW cotton production areas (marked by blue polygons). **a**) Historical climate suitability across NSW, **b**) Future climate suitability under the intermediate emissions scenario RCP4.5, **c**) change in climate suitability under RCP4.5, **d**) confidence in change under RCP4.5. **e**) Future climate suitability under the high emissions scenario RCP8.5, **f**) change in climate suitability under RCP8.5, **g**) confidence in change under RCP8.5

Historically, climate suitability in these northern regions of NSW has been high. However, future projections indicate potential increases in climate suitability, particularly in the early part of the cotton growing season (September & October). Figure 5 shows that Moree, Narrabri, Bourke and Warren are likely to experience increased climate suitability at planting time. The October maps (Figure 6) demonstrate climate suitability at a common planting time for cotton, although many growers plant earlier in September or later in November, depending on favourable weather conditions.

By 2050, climate suitability in these northern cotton-growing regions during summer months (December, January, February) is projected to increase. This increase is expected to be more pronounced under the high emissions scenario. The October maps (Figure 6) illustrate this increase in climate suitability, particularly in the north of the state, under the high emissions scenario (Figure 6e and f). This change could increase the potential for infection and survival of the defoliating strain. In the southern regions of Hillston and Griffith, climate suitability for the D strain is likely to increase minimally during autumn (March, April, May) and spring (September, October, November) under the high emissions scenario.

### Dormant life stage

**Fig. 7:**
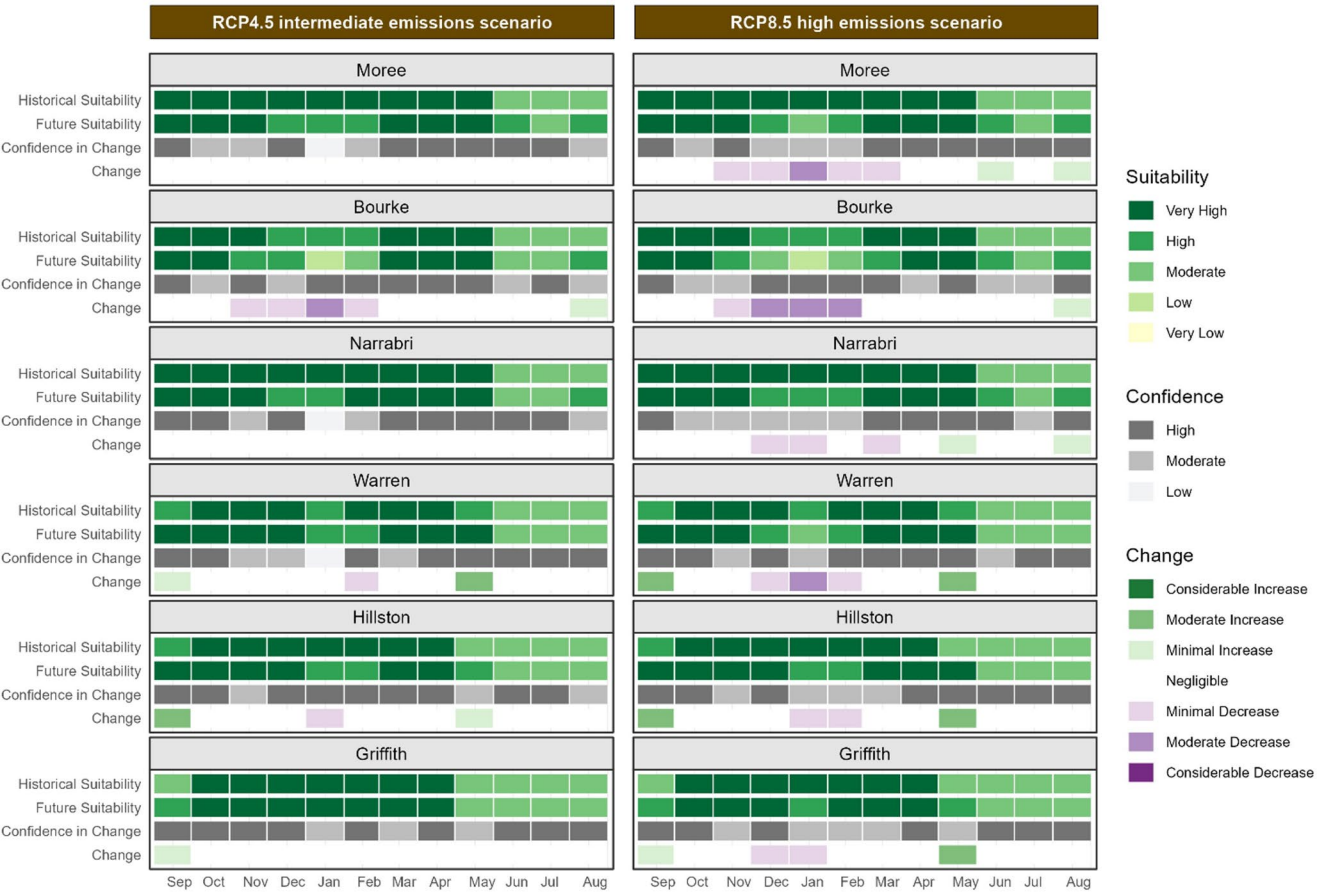
Calendar plot for dormant life stage of *V. dahliae* during the cotton season in key cotton growing regions displaying the historical and future climate suitability, change in suitability and confidence in change for each site of interest. Two emissions scenarios are shown: the intermediate emissions scenario, RCP4.5, on the left and the high emissions scenario, RCP8.5, on the right. The climate suitability categories represent the median of values within a 10 km radius of each location

**Fig. 8:**
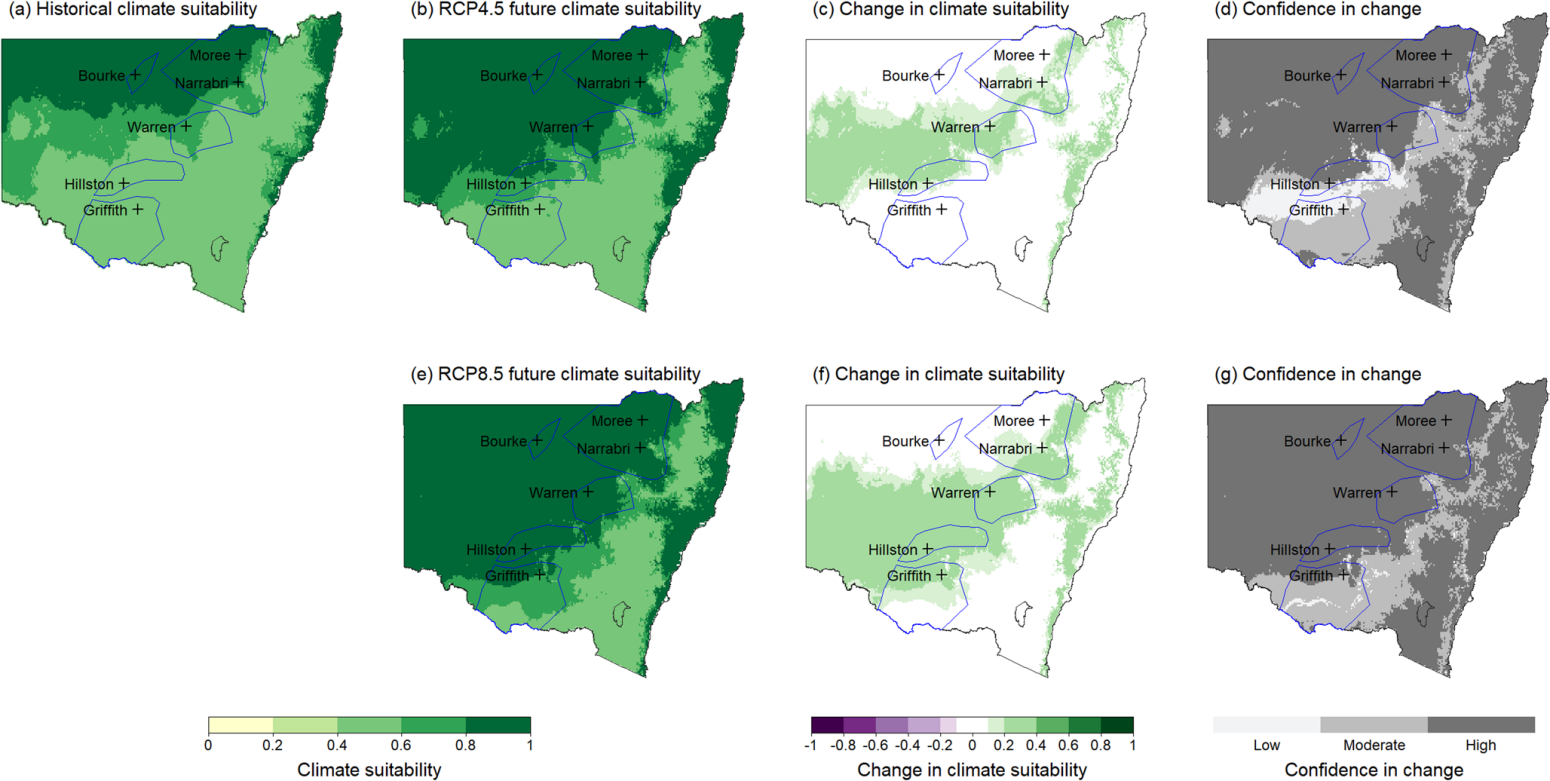
Map for May showing potential changes in climate suitability for the dormant life stage of *V. dahliae* across NSW cotton production areas (marked by polygons). **a**) Historical climate suitability across NSW, **b**) Future climate suitability under the intermediate emissions scenario RCP4.5, **c**) change in climate suitability under RCP4.5, **d**) confidence in change under RCP4.5. **e**) Future climate suitability under the high emissions scenario RCP8.5, **f**) change in climate suitability under RCP8.5, **g**) confidence in change under RCP8.5

The climate impact modelling projects changes in the suitability of *V. dahliae* dormant life stage across NSW cotton growing regions by 2050. These changes vary by season, location, and emissions scenario, as illustrated in Figs.7 and 8. Seasonal trends indicate a general increase in climate suitability during autumn, winter, and spring for most cotton growing regions under both emissions scenarios. Conversely, climate suitability in summer is projected to decrease, with more pronounced reductions under the high emissions scenario.

Historically, the dormant life stage has not faced climatic restrictions in the northern cotton growing regions Moree, Bourke, and Narrabri from September through May; climate suitability in these areas has been high to very high. By 2050, these areas are likely to experience minimal to moderate reductions in climate suitability in the warmer summer months, particularly under the high emissions scenario at Moree and Bourke. Some minimal increases in climate suitability are likely during the cooler months at all northern sites.

The southern cotton growing regions, Warren, Hillston, and Griffith have historically had high to very high climate suitability for the dormant life stage from October to April. By 2050, the warmer summer months are projected to decrease in suitability, particularly under the high emissions scenario. However, the cooler months of May and September are likely to see minimal increases in climate suitability for all sites to either high or very high suitability by 2050. The high to very high suitability in September in the southern cotton growing regions could increase the survival of microsclerotia in the soil, increasing the risk of infection if planting occurs in September. Additionally, the increased suitability in May (shown in Figure 8), following harvest when microsclerotia have been returned to the soil increases the risk of survival and overwintering prior to planting the following crop.

## Discussion

The results of this study provide valuable insights into the historical and potential future climate suitability of different life stages and strains of *V. dahliae*, with important implications for cotton-growing regions in NSW. The climate suitability maps, and calendar plots depict the variations in suitability across different months and regions for each life stage of the pathogen. Our findings reveal climate suitability shifts during various months corresponding to the parasitic life stages of the ND and D strains and the dormant life stage, which is significant to Australian cotton production. However, the effects of Verticillium wilt disease in cotton will depend on the future distribution of cotton growing in NSW, changes in production timings, the overlap between susceptible life stages of cotton, the lifecycle of *V. dahliae* and the management strategies used to minimise the disease.

Under climate change, the distribution and abundance of the ND and D strains and the survival of microsclerotia during the dormant life stage is likely to change in NSW. One of the key observations is the consistently high climate suitability during the cotton growing season (September to April) in key cotton growing regions. For instance, the ND strain demonstrated high suitability in regions like Moree and Narrabri during the growing season. Under the high emissions scenario, the minimal to moderate increase in suitability for the ND strain may occur across inland NSW in October. The Narrabri cotton growing region may see an increase in disease as this period aligns with optimal pathogen conditions for infection at the time of planting in October.

Cotton in the northern growing regions of NSW could be particularly affected due to increased climate suitability for the D strain, potentially leading to increased yield losses due to the disease. Moderate suitability for the D strain occurs in northern NSW in spring and summer. This change could present an increased risk for cotton production as the timing of increased suitability for the D strain correlates with the planting dates for the northern cotton growing region. Early infection with the defoliating strain can cause severe defoliation, yield losses and plant death.

Climate suitability for the dormant life stage is high to very high and is likely to increase in most cotton growing regions in NSW during the cooler months. Future climate change is unlikely to prevent the microsclerotia forming in the dying and dead cotton material in winter. Increased suitability during winter is particularly important in relation to host weeds growing during winter expanding into different regions. Warming temperatures are likely to lengthen the cotton growing season of cotton, increasing plant biomass, and causing a shorter period for decomposition of trash. Higher winter temperatures could increase the survival of microsclerotia on overwintering crop residues and increase the inoculum available to infect subsequent crops (Coakley et al. 1999).

Management strategies for controlling volunteer cotton and weeds will become more important as these provide a green bridge between crops. Cotton varieties are assigned a disease rank provided by Cotton Seed Distributors using an industry developed protocol that provides a quantitative measure of the relative resistance or susceptibility of cotton varieties to Verticillium wilt (Cotton Seed Distributors 2024). This ranking is referred to as V-rank. In Australia, cotton varietal resistance to Verticillium wilt is temperature sensitive, meaning cotton varieties with a high V-rank that are resistant at 25–27 °C will succumb to disease when average temperatures drop to 20–22 °C or below (Holman et al. 2016). Given that Verticillium wilt resistance in these varieties is temperature sensitive and the different climate suitability of each strain, plant breeding for resistance/tolerance to each strain will be a priority.

The ND strain is present throughout NSW, as reported in the NSW cotton disease surveys (Le et al. 2020; while the D strain is more prevalent only in the northern cotton growing regions of NSW. With yield losses associated with both strains, and the occurrence of coinfection, management of this disease will remain a priority for the industry. Redistribution of strains due to changes in climate suitability may increase the prevalence the D strain in the northern cotton growing regions of NSW.

The impact of *V. dahliae* VCGs in other important agricultural crops is not well understood, however the disease has the capacity to cause significant economic losses. In 2022, a VCG6 isolate was reported for the first time in Noogoora burr (*Xanthium occidentale*) in NSW (Kirkby et al. 2022), but the effect on cotton and other crops has not been reported. Analysis of the future climate projections indicates a potential change in the number of months that are highly suitable for *V. dahliae*, leading to a shift in strain distribution in NSW, with the expected prevalence of the D strain in northern NSW and ND strain becoming more prevalent in the state’s southern regions. The implications of climate suitability range from detrimental to positive outcomes, ultimately shaping the trajectory of the cotton industry in the coming years. Understanding the potential for redistribution of strains in the different cotton growing regions provides an insight into the management of Verticillium wilt and a need for the cotton plant breeding program to select varieties for tolerance/resistance to both of the two strains.

## Conclusion

This study provides critical insights into the historical and projected climate suitability of *V. dahliae* in cotton-growing regions of NSW, highlighting significant shifts in the distribution and prevalence of the ND and D strains due to climate change. Our findings indicate that the northern regions of NSW, particularly Bourke, Moree, and Narrabri, are likely to experience an increased suitability for the D strain, which could pose greater risks for cotton production, especially during planting periods aligned with optimal pathogen conditions. Conversely, while the ND strain may see a moderate decline in climate suitability in these areas, the southern regions of Hilston and Griffith are expected to maintain a stable environment for its growth and survival.

As climate conditions evolve, the survival and infectivity of *V. dahliae* will be influenced not only by temperature and rainfall changes, but also by management practices that mitigate the risks posed by this pathogen. The historical resilience of certain regions may be challenged as warmer autumns and winters create a conducive environment for microsclerotia survival and subsequent infection. Consequently, proactive management strategies, including the cultivation of resistant cotton varieties to each strain and careful timing of planting, will be essential to mitigate the impact of Verticillium wilt on cotton yield and quality.

This MCA model is a unique and cost effective method used to understand the potential complex impacts of climate change on the pathogen that causes Verticillium wilt in cotton and many other agronomically important crops. This MCA model was prepared to examine the effect of climate change on *V. dahliae* in NSW, however it could be expanded to other states where cotton is grown such as QLD. Additionally, it could be used to model the impact of changing climates in other countries on *V. dahliae* and other pathogens of interest.

The MCA model relies heavily on published literature and research data to define the climate variables and the importance of these variables. In preparing the information required, gaps in the scientific literature became apparent and were overcome by including expert knowledge from scientific experts. Gaps were recorded in a gap analysis, which provided a document that can be used to direct future research requirements.

In summary, this research underscores the need for an adaptive approach to cotton cultivation in NSW, informed by climate future projections and pathogen behaviour. By anticipating changes in strain distribution and their implications for NSW cotton growers, stakeholders can implement targeted strategies to ensure the sustainability of the industry’s future. As climate change continues to reshape agricultural landscapes, ongoing monitoring and research including the impact of climate change on the other five species of Verticillium will be vital to ensure the resilience and sustainability and preparedness of cotton production in NSW and beyond.

## Supplementary Information

Below is the link to the electronic supplementary material.

Supplementary Material 1 (17.5 KB)

Supplementary Material 2 (37.5 KB)

Supplementary Material 3 (30.2 KB)

## Data Availability

The datasets are being prepared as part of a larger data release. Register your interest with vulnerability.assessment@dpi.nsw.gov.au, and when the data is available, you’ll be notified.
