# Supplementary material for: Assessing the impact of climate change on verticillium wilt and the implications for cotton production in Australia
Source: Int J Biometeorol. 2026 Feb 10;70(2):57. doi: 10.1007/s00484-025-03100-5 (PMC12891031; doi:10.1007/s00484-025-03100-5)
Supplement: Supplementary file 1 — Supplementary Material 1 (17.5 KB) [file 484_2025_3100_MOESM1_ESM.docx]

**Supplementary Table 1.** Peer-reviewed journal articles used to inform the MCA hierarchy for life stages and climate variables in the MCA model development.

| **No.** | **References** | **Life stage** | **Variable** |
| --- | --- | --- | --- |
| 1 | Calderón, R., et al. (2014). “Soil Temperature Determines the Reaction of Olive Cultivars to *Verticillium dahliae Pathotypes*.” PLos ONE **9**(10 e110664). | Parasitic (ND) & Dormant | Temperature |
| 2 | Chakraborty, S., et al. (2000). “Climate change: potential impact on plant diseases.” Environmental Pollution **108**: 317-326. | Parasitic & Dormant | Temperature |
| 3 | Devaux, A. L. and W. E. Sackston (1966). “Taxonomy of Verticillium species causing wilt of horticultural crops in Quebec.” Canadian Journal of Botany **44**: 803-811. | Parasitic (ND & D) & Dormant | Temperature & Rainfall |
| 4 | Jabnoun-Khiareddine, H., et al. (2006). “Effect of Temperature on Verticillium Wilts of Tomato in Tunisia.” Plant Pathology Journal **5**: 1-6. | Parasitic (ND) | Temperature |
| 5 | Jiménez-Diaz, R. M., et al. (2012). “Verticillium wilt, a major threat to olive production: Current Status and Future Prospects for its Management.” Plant Disease **96**(3): 304-329. | Parasitic (ND & D) & Dormant | Temperature |
| 6 | Landa, B. B. (2019). “Focus Groups on Pests and Diseases of Olive Tree – Starting Paper.” from <https://ec.europa.eu/eip/agriculture/en/publications/eip-agri-focus-group-pests-and-diseases-olive-tree>. | Parasitic (ND & D) | Temperature |
| 7 | Leyendecker, P. J. (1950). “Effects of certain cultural practices on Verticillium wilt of cotton in New Mexico.” Bulletin of the New Mexico Agricultural Experimental Station: 356. | Parasitic (ND & D) | Pathotype & Rainfall |
| 8 | Ludbrook, M. V. (1932). “Pathogenicity and environmental studies on *Verticillium hadromycosis*.” Phytopathology **23**: 117-154. | Parasitic (ND & D) & Dormant | Temperature & Rainfall |
| 9 | Morello, P., et al. (2016). “Sanitation of olive plants infected by *Verticillium dahliae* using heat treatments.” Plant Pathology **65**: 412-421. | Dormant | Temperature |
| 10 | Mozumder, B. K. G., et al. (1970). “Influence of water activity, temperature and their interaction germination of *Verticillium albo-atrum* conidia. .” Plant Physiology **46**: 347-349. | Parasitic (ND & D) | Pathotype & Temperature |
| 11 | Pegg, G. F. and B. L. Brady (2002). Verticillium wilts. Wallingford, UK, CABI Publishing. | Parasitic (ND & D) | Temperature |
| 12 | Puhalla, J. E. and J. E. Mayfield (1974). “The mechanisms of heterokaryotic growth in *Verticillium dahliae*.” Genetics **76**: 411-422. | Dormant | Temperature |
| 13 | Schnathorst, W. C., et al. (1975). “*Verticillium dahliae* strains in cotton in the Pahrump valley, Nevada. .” Plant Disease Reporter **59**: 863-865. | Parasitic (D) | Temperature |
| 14 | Schneider, H. (1948). “Susceptibility of guayule to Verticillium wilt and influence of soil temperature and moisture on development of infection.” Journal of Agricultural Research **76**: 129-143. | Parasitic (D) | Temperature |
| 15 | Soesanto, L. and A. J. Termorshuizen (2013). “The effect of temperature on the formation of microsclerotia of *Verticillium dahliae*.” Journal of Phytopathology **149**: 11-12. | Dormant | Temperature |
| 16 | Subbarao, K. V., et al. (1995). “Genetic relationships and cross pathogenicities of *Verticillium dahliae* isolates from cauliflower and other crops.” Phytopathology **85**: 1105-1112. | Parasitic (ND) & Dormant | Temperature & Rainfall |
| 17 | Subbarao, K. V. and J. C. Hubbard (1996). “Effects of broccoli residue and temperature on *Verticillium dahliae* microsclerotia in soil and on wilt in cauliflower.” Phytopathology **86**: 1303-1310. | Parasitic (ND & D) | Pathotype |
| 18 | Wyllie, T. D. and J. E. DeVay (1970). “Growth characteristics of several isolates of *Verticillium albo-atrum* and *Verticillium nigrescens* from cotton.” Phytopathology **60**: 907-910. | Parasitic (ND & D) & Dormant | Temperature & Rainfall |
