# Supplementary material for: Assessing the impact of climate change on verticillium wilt and the implications for cotton production in Australia
Source: Int J Biometeorol. 2026 Feb 10;70(2):57. doi: 10.1007/s00484-025-03100-5 (PMC12891031; doi:10.1007/s00484-025-03100-5)
Supplement: Supplementary file 2 — Supplementary Material 2 (37.5 KB) [file 484_2025_3100_MOESM2_ESM.docx]

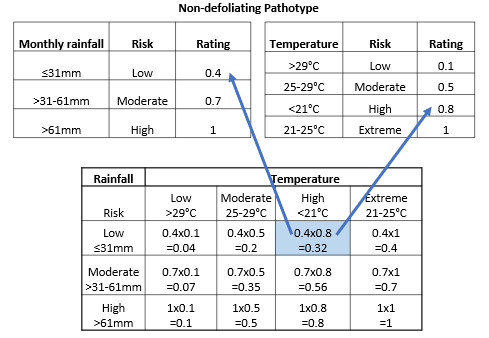


**Supplementary Figure 1.** Schematic showing the calculation for ratings in the non-defoliating strain matrix.
