# Supplementary material for: Assessing the impact of climate change on verticillium wilt and the implications for cotton production in Australia
Source: Int J Biometeorol. 2026 Feb 10;70(2):57. doi: 10.1007/s00484-025-03100-5 (PMC12891031; doi:10.1007/s00484-025-03100-5)
Supplement: Supplementary file 3 — Supplementary Material 3 (30.2 KB) [file 484_2025_3100_MOESM3_ESM.docx]

**Supplementary Table II.** Rainfall and temperature categories used to calculate the interaction ratings for both ND and D strains. Note temperature differences between strains. Rainfall and temperature categories and ratings for the dormant life stage. Interaction ratings (for rainfall and temperature) in the matrix for the parasitic life stage were calculated by multiplying the mean temperature ratings in each category with the mean rainfall ratings (low, medium, and high) within each category.

| **Parasitic Life Stage** | | | | | | |  | **Dormant life stage** | | |
| --- | --- | --- | --- | --- | --- | --- | --- | --- | --- | --- |
| **Non-defoliating Strain** | | |  | **Defoliating Strain** | | |  |  |  |  |
| **Temperature** | **Risk** | **Rating** |  | **Temperature** | **Risk** | **Rating** |  | **Temperature** | **Risk** | **Rating** |
| >29°C | Low | 0.1 |  | ≤20°C | Low | 0.1 |  | ≥30°C | Low | 0.1 |
| 25-29°C | Medium | 0.5 |  | 20-24°C | Medium | 0.5 |  | <15°C | Medium | 0.5 |
| <21°C | High | 0.8 |  | >28°C | High | 0.8 |  | 25-30°C | High | 0.8 |
| 21-25°C | Extreme | 1 |  | 24-28°C | Extreme | 1 |  | 15-25°C | Extreme | 1 |
|  |  |  |  |  |  |  |  |  |  |  |
| **Monthly rainfall** | **Risk** | **Rating** |  | **Monthly rainfall** | **Risk** | **Rating** |  | **Monthly rainfall** | **Risk** | **Rating** |
| ≤31mm | Low | 0.4 |  | ≤31mm | Low | 0.4 |  | ≤31mm | Low | 0.7 |
| >31-61mm | Medium | 0.7 |  | >31-61mm | Medium | 0.7 |  | >31-61mm | Medium | 0.85 |
| >61mm | High | 1 |  | >61mm | High | 1 |  | >61mm | High | 1 |
